# The Study of Angptl4-Modulated Podocyte Injury in IgA Nephropathy

**DOI:** 10.3389/fphys.2020.575722

**Published:** 2021-02-11

**Authors:** Sha Jia, Xiaofeng Peng, Ludan Liang, Ying Zhang, Meng Li, Qin Zhou, Xiujin Shen, Yucheng Wang, Cuili Wang, Shi Feng, Jianghua Chen, Pingping Ren, Hong Jiang

**Affiliations:** ^1^Kidney Disease Center, The First Affiliated Hospital, College of Medicine, Zhejiang University, Hangzhou, China; ^2^Key Laboratory of Nephropathy, Hangzhou, China; ^3^Kidney Disease Immunology Laboratory, The Third-Grade Laboratory, State Administration of Traditional Chinese Medicine of China, Hangzhou, China; ^4^Key Laboratory of Multiple Organ Transplantation, Ministry of Health of China, Beijing, China; ^5^Institute of Nephropathy, Zhejiang University, Hangzhou, China; ^6^Dongyang Women & Children Hospital, Dongyang, China

**Keywords:** Angptl4, podocyte, immunoglobulin A nephropathy, RNA-Seq, progression

## Abstract

**Background:**

Increasing evidence shows that Angptl4 affects proteinuria in podocytes injured kidney disease, however, whether there is a relationship between Angptl4 and IgA nephropathy (IgAN) has not been studied yet.

**Methods:**

Plasma and urine samples were obtained from 71 patients with IgAN and 61 healthy controls. Glomeruli from six renal biopsy specimens (three IgAN patients and three healthy controls) were separated by RNA-Seq. Differentially expressed genes (DEGs) related to podocytes and Angptl4 between IgAN patients and healthy controls were performed using the Limma package. Gene set enrichment analysis was used to determine whether there was a statistically significant difference between the two groups. STRING was used to create a protein-protein interaction network of DEGs. Association analysis between Angptl4 levels and clinical features of IgAN was performed.

**Results:**

Thirty-three podocyte-related and twenty-three Angpt4-related DEGs were found between IgAN patients and healthy controls. By overlapping the genes, *FOS* and *G6PC* were found to be upregulated in IgAN patients, while *MMP9* was downregulated in IgAN patients. Plasma and urine Angptl4 levels were closely related to the degree of podocyte injury and urine protein, but not to the protein-creatine ratio.

**Conclusion:**

Our findings show that Angptl4 levels in plasma and urine are related to podocyte damage and, therefore, may be a promising tool for assessing the severity of IgAN patients to identify and reverse the progression to ESRD.

## Introduction

Immunoglobulin A nephropathy (IgAN), which is characterized by galactose-deficient IgA deposits in the glomerular mesangium, is the most prevalent type of glomerulonephritis worldwide (Suzuki et al., 2011; Wyatt and Julian, 2013). IgAN is one of the major causes of end-stage renal disease (ESRD), proteinuria, and hypertension. Reduced glomerular filtration rates are often used to assess its prognosis in clinical practice (Floege and Feehally, 2013). Nevertheless, a new indicator that would provide meaningful information about the diagnosis of IgAN and the effects of its treatment is still needed.

It is generally thought that mesangial cells play a dominant role in the pathogenesis of IgAN, but since mesangial-podocytic-tubular crosstalk has been proposed, emerging evidence shows that the role of podocytes in IgAN should not be underestimated (Hishiki et al., 2001; Faul et al., 2007; Lai, 2012; Fukuda et al., 2015; Leung et al., 2018). Podocytes possess numerous foot processes, surround glomerular capillaries, and serve as the last barrier to renal filtration (Greka and Mundel, 2012). Foot process effacement is a hallmark of podocyte injury, which leads to proteinuria and glomerulosclerosis (Lemley et al., 2002; Hara et al., 2007).

Angiopoietin-like protein 4 (Angptl4), an inhibitor of lipoprotein lipase (Yoshida et al., 2002), has been the focus of many studies examining novel mechanisms of proteinuria in recent years (Clement et al., 2011, 2014; Li et al., 2015; Ma et al., 2015). A previous study indicated that increased secretion of Angptl4 with a high isoelectric point (pI) by podocytes leads to proteinuria and foot process effacement in humans, and experimentally, minimal change disease (MCD) (Clement et al., 2011). However, circulating Angptl4 with a neutral pI, which is mainly secreted by adipose tissue and liver, reduces proteinuria by binding to αvβ5 integrin on the glomerular endothelium (Clement et al., 2014). Our previous study also observed an important role of Angptl4 in podocyte injury-associated nephropathy (Chen et al., 2013). However, the changes in Angptl4 expression levels in IgAN have not yet been investigated.

To more easily diagnose and evaluate the severity of IgAN, it is vital to find novel and non-invasive biomarkers. In this study, we attempted to use RNA sequencing of glomeruli separated from biopsy tissues of IgAN patients and healthy controls to explore the potential functions of Angptl4 in IgAN. We further confirmed the relationship between Angptl4 expression and IgAN in clinical samples.

## Materials and Methods

### Study Population

All procedures in this study involved human participants in accordance with the Declaration of Helsinki. The study was approved by the Ethical Committee of the Zhejiang University College of Medicine, First Affiliated Hospital. All subjects (patients and healthy controls) provided written informed consent for blood, urine, or tissue collection. Seventy-one adult patients with biopsy-proven idiopathic IgAN were included in the IgAN group, and 60 live renal transplant donors were included in the healthy control group. The IgAN group was further divided into two groups according to electron microscopy results: one group consisted of 37 patients with foot process fusion, and the other group consisted of 34 patients without foot process fusion.

### Tissue Samples

Plasma and urine samples were collected at the time of kidney biopsy before treatment. Laser-captured microdissected glomeruli were obtained from the kidney biopsy tissues of three IgAN patients and three healthy controls for RNA sequencing. Dissection of the glomeruli under the microscope was performed according to a previously described protocol (Jiang et al., 2008). In short, the glomeruli were dissected with sharp forceps under a stereoscopic electron microscope. The small tubules, except for the medullary thick ascending limb of Henle’s loop, were removed. All the steps were carried out in an albumin-rich saline solution (0.1%) at 4°C. Dissection was completed within 120 min of the kidney biopsy. The studies on human samples were conducted according to the Declaration of Helsinki.

### Library Preparation for Sequencing

RNA was extracted from the glomeruli after dissection. The RNA concentration and purity were determined using a Qubit^®^ 2.0 fluorometer (Qubit^®^ RNA Analysis Kit, Life Technologies, California, United States) and NanoPhotometer^®^ (Implen, California, United States), respectively. A 1% agarose gel was used to assess RNA degradation and contamination. The RNA Nano 6000 Assay Kit of the Agilent Bioanalyzer 2100 system (Agilent Technologies, CA, United States) was used to assess RNA integrity.

A total of 3 μg of RNA per sample was used as the input material for the RNA sample preparations. The sequencing libraries were generated using the NEBNext^®^ Ultra^TM^ RNA Library Prep Kit for Illumina^®^ (NEB, United States) following the manufacturer’s instructions, and index codes were added to label the sequences of each sample. Briefly, mRNA was purified from total RNA using poly T oligo-conjugated magnetic beads. Fragmentation was conducted using divalent cations under an elevated temperature in NEBNext First-Strand Synthesis Reaction Buffer (five times). Synthesis of the first-strand and second-strand cDNA was carried out using a random hexamer primer, M-MuLV Reverse Transcriptase (RNaseH-), DNA Polymerase I, and RNase H. The remaining overhangs were converted to blunt ends through exonuclease/polymerase activities. After the 3′ terminus of the DNA fragments was adenylated, ligation of the NEBNext adaptor with a hairpin loop structure was performed to prepare for hybridization. To select cDNA fragments of 150–200 bp in length, the library fragments were purified with the AMPure XP system (Beckman Coulter, Beverly, MA, United States). Then, 3 μL of USER Enzyme (NEB, United States) was incubated with size-selected and adaptor-ligated cDNA at 37°C for 15 min, followed by 5 min at 95°C before PCR. Then, PCR was carried out with Phusion High-Fidelity DNA polymerase, universal PCR primers, and Index (X) Primer. Finally, the PCR products were purified on the AMPure XP system, and the library quality was assessed using the Agilent Bioanalyzer 2100 system.

### Clustering and Sequencing (Novogene Experimental Department)

The clustering of the index-coded samples was carried out on a cBot Cluster Generation System with the TruSeq SR Cluster Kit v3-cBot-HS (Illumina). All processes were conducted according to the manufacturer’s instructions. The sequencing of the library was conducted on the basis of an Illumina HiSeq 2000/2500 platform to generate 150/100/50 bp paired/single-end reads.

### Bioinformatics Analysis

FastQC and Trimmoatic were used to assess the quality of the raw sequencing reads and to clean the sequencing adaptor sequences at both ends of the raw reads. Clean reads were mapped using STAR to the human genome hg19. Gene expression was calculated, and the identification of differentially expressed genes (DEGs) was conducted using Cuffdiff 2 with a *q*-value < 0.05. Functional annotation and pathway enrichment analysis with DAVID were conducted. Polysearch2 and another web server that supports text mining from Medline and PubMed were used to search for Angptl4-related or podocyte-related genes. After the extraction of the related genes, a relevance score was calculated and converted to a Z-score. Then, the genes with *Z*-scores above 1 were selected for subsequent analysis. IgAN-related genes were searched by literature mining from the PubMed database.

### Measurement of the Concentration of Angptl4 in Plasma and Urine From Patients With IgAN

Plasma and urine samples were collected from IgAN patients and enzyme-linked immunosorbent assay (ELISA) was used to determine and compare the concentration of Angptl4 in the different samples. The characteristics of Angptl4 expression were analyzed and combined with the electron microscopy observation results and urinary protein levels.

### ELISA

The Angptl4 levels in plasma and urine were analyzed using commercial immunoassay kits (Angiopoietin-Like protein 4 Human ELISA, BioVendor, Human IgG4 Platinum ELISA, eBioscience) according to the manufacturer’s instructions.

### Measurement of Podocyte Injury by Electron Microscopy

Renal cortical and medullary tissues from IgAN patients and healthy controls were minced into 1 mm^3^ pieces and processed for electron microscopy using standard protocols. Ultrathin sections (80–90 nm) were prepared for examination and imaging with an Olympus transmission electron microscope (Tecnai, Tokyo, Japan).

### Statistical Analyses

Statistical analyses were carried out using GraphPad Prism software (version 8.3.0). All data are presented as the mean ± SEM. Analysis of the differences in Angptl4 expression was conducted by one-way ANOVA with Dunnett’s *post hoc* test using GraphPad. The one-sample Kolmogorov–Smirnov test was used to assess for normal distribution of the data. Relationships between the Angptl4 levels and clinical parameters were examined using Pearson correlation analysis when normally distributed; otherwise, the Spearman rank test was used. All *P*-values were two-tailed, and significance was defined as *P* < 0.05.

## Results

### Demographic Characteristics of the IgAN Patients and Healthy Controls

The clinical data of the patients and controls are shown in Table 1. Clinical parameters, such as serum albumin (g/L), serum creatinine (μmol/L), 24-hour urine protein excretion (g/day), and protein-creatinine ratio (g/g), were examined at the time of renal biopsy. No significant differences in age or sex were observed among the three groups. The patients in the IgAN with and without podocyte injury groups presented with a mean serum creatinine level of 187.22 ± 20.61 and 95.94 ± 5.97 μmol/L, and a mean serum albumin level of 35.20 ± 0.98 and 37.89 ± 1.25 g/L, respectively (*P* < 0.05) compared with the healthy controls). The controls had no proteinuria and presented with a mean serum albumin level of 44.26 ± 0.26 g/L.

**TABLE 1:** Characteristics of individuals included in the study.

	IgAN		
	IgAN with podocyte injury (*n* = 37)	IgAN without podocyte injury (*n* = 34)	Control (*n* = 61)
Age (years)	42.89 ± 1.78	45.44 ± 2.15	45.49 ± 1.29
Sex (male/female)	23/14	20/14	45/16
Serum albumin (g/L)	35.20 ± 0.98*	37.89 ± 1.25*	44.26 ± 0.26
Scr (μmol/L)	187.22 ± 20.61*	95.94 ± 5.97*	72.92 ± 1.30
24 UP (g/day)	3.06 ± 0.40	1.35 ± 0.48	
P/C (g/g)	1.23 ± 0.26	1.60 ± 0.35	
Oxford evaluation			
M1	12	7	
E1	2	3	
S1	32	20	
T1 or T2	11	5	

Scr, serum creatinine; 24 UP, 24-hour urine protein excretion; P/C, protein: creatinine ratio. The data are shown as the mean ± SEM. *P < 0.05 versus the control.

### Gene Expression Analysis by RNA-Seq

**FIGURE 1:**
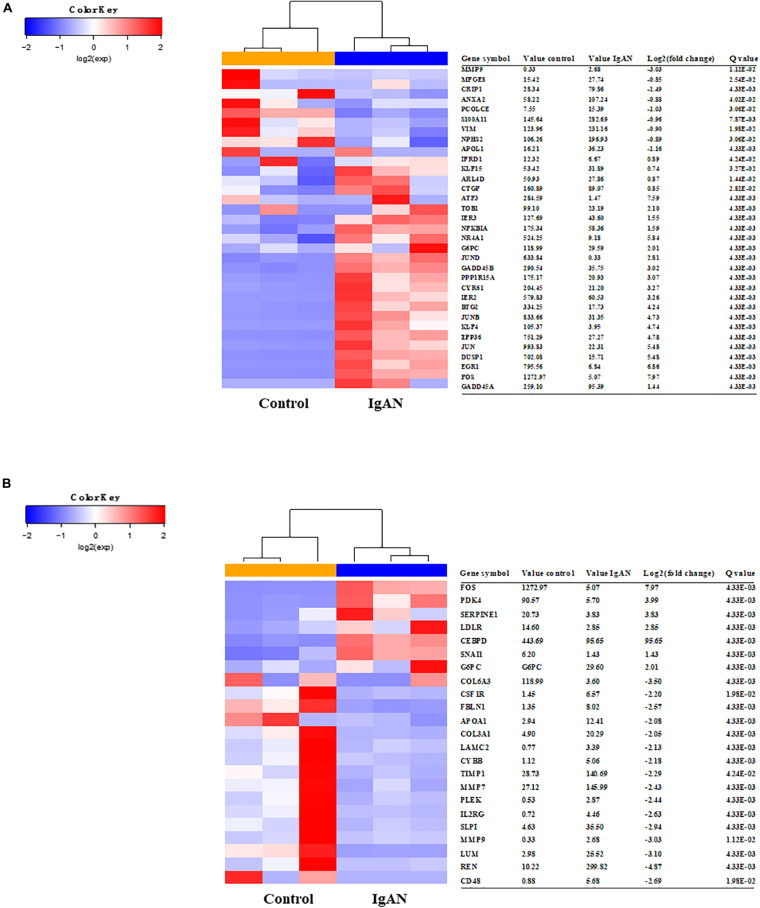
Heatmaps of differentially expressed genes associated with Angiopoietin-like protein 4 and podocyte injury in IgA nephropathy (IgAN) patients compared to healthy controls. **(A)** The glomerular expression levels of 33 differentially expressed genes that were previously reported to be related to podocytes. The red areas represent genes expressed at high levels and the blue areas represent genes expressed at low levels in IgAN patients compared with healthy controls. The official gene names and test values of the IgAN patients and the controls are provided. A positive log_2_ (fold change) indicated upregulation in the disease group, and a negative log_2_ (fold change) indicated downregulation in the disease group. **(B)** The glomerular expression levels of 23 differentially expressed genes correlated with Angptl4. Refer to panel **(A)** for the description of the figure.

We used STRING to analyze protein interactions and screened DEGs that were directly or indirectly related to Angptl4. The red and blue colors in the heatmap depict higher and lower gene expression, respectively. The official gene names are given, the test values of the IgAN patients and the controls are listed. The fold changes indicate the relative alteration in the IgAN value compared to the control value, and the log_2_ value of the fold change is shown. A positive log_2_ (fold change) indicated upregulation in the disease group, and a negative log_2_ (fold change) indicated downregulation in the disease group. The glomerular expression levels of 33 transcripts related to podocytes and 23 transcripts correlated with Angptl4 (Figures 1A,B).

### Gene Set Enrichment Analysis of Angptl4

**FIGURE 2:**
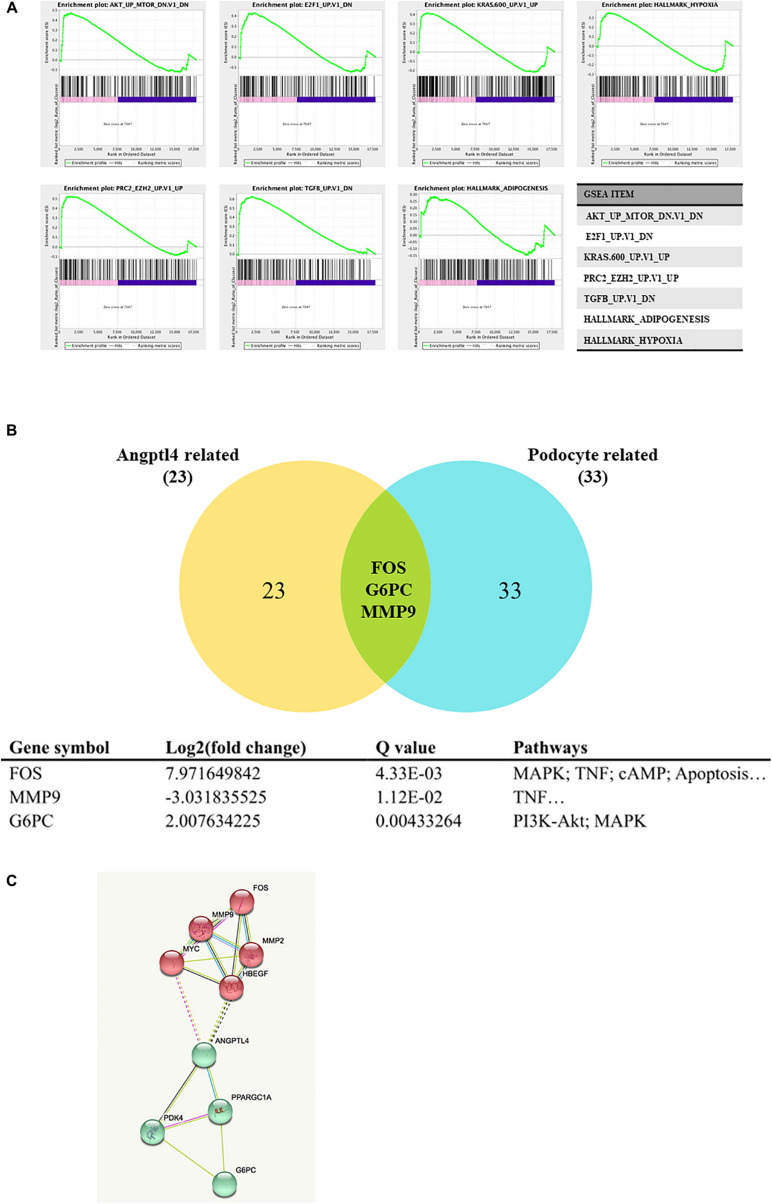
Gene set enrichment analysis of Angiopoietin-like protein 4. **(A)** The Angptl4 gene participated in 7 enriched sets that were statistically significantly different between the IgA nephropathy (IgAN) group and the control group. **(B)** The transcripts of three genes, namely, *FOS*, *G6PC*, and *MMP9*, were simultaneously included in the Angptl4-related and podocyte-related genes. Two of these genes, namely, *FOS* and *G6PC*, were upregulated in the disease group, whereas *MMP9* was downregulated in the disease group. **(C)** The depiction of the network of Angptl4, *FOS*, *G6PC*, and *MMP9* with several differentially expressed genes selected from among the Angptl4-related or podocyte-related genes by STRING.

GSEA 3.0 software was used to analyze the Angptl4 gene enrichment results. The Angptl4 gene participated in seven enriched sets, which were statistically significantly different between the IgAN group and the control group. The transcripts of three genes, FOS, G6PC, and MMP9, were simultaneously included in the Angptl4-related and podocyte-related genes (Figure 2A). Two of these genes, namely, FOS and G6PC, were upregulated in the disease group, whereas MMP9 was downregulated in the disease group (Figure 2B). STRING was used to depict the protein-protein interaction network of Angptl4, FOS, G6PC, and MMP9 with several DEGs selected from among the Angptl4-related or podocyte-related genes (Figure 2C).

### Angptl4 in Plasma and Urine

**FIGURE 3:**
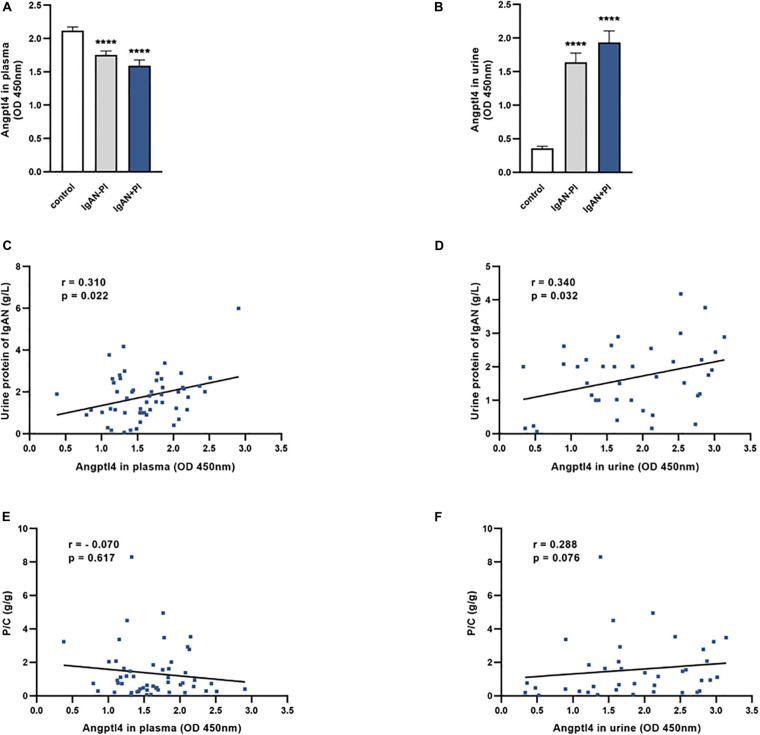
Angiopoietin-like protein 4 levels in plasma and urine. **(A,B)** The expression of Angptl4 in plasma and urine was detected in IgA nephropathy (IgAN) without podocyte injury patients (*n* = 34), IgAN with podocyte injury patients (*n* = 37), and normal controls (*n* = 61) by enzyme-linked immunosorbent assay. **(C,D)** The relationship between urine protein (g/L) and Angptl4 expression in plasma and urine was analyzed in IgAN patients (*n* = 61). **(E,F)** The relationship between the urine protein-creatinine ratio (g/g) and Angptl4 expression in plasma and urine was analyzed in IgAN patients (*n* = 61). The data are expressed as the mean ± SEM. ***P < 0.001 compared with the normal controls.

Plasma Angptl4 levels were significantly lower in IgAN patients than in normal controls. The expression of Angptl4 in plasma was decreased in the IgAN without podocyte injury patients compared with the normal controls (1.75 ± 0.06 versus 2.12 ± 0.05, ****P < 0.0001). The plasma Angptl4 level in the IgAN with podocyte injury patients was also significantly lower than that in the controls (1.59 ± 0.09 versus 2.12 ± 0.05, ****P < 0.0001). There were no significant differences between the IgAN without podocyte injury and IgAN in podocyte injury patients (Figure 3A). The Angptl4 expression level in urine was significantly higher in IgAN patients than in normal controls. Urine Angptl4 expression was increased in the IgAN without podocyte injury patients compared with the normal controls (1.64 ± 0.14 versus 0.36 ± 0.03, ****P < 0.0001). The urine Angptl4 level in the IgAN with podocyte injury patients was also significantly higher than that in the controls (1.93 ± 0.17 versus 0.36 ± 0.03, ****P < 0.0001). Similarly, no significant difference was observed between the IgAN without podocyte injury and IgAN in podocyte injury patients (Figure 3B). In the IgAN patients, there was a significant correlation between the levels of urine protein and the levels of plasma Angptl4 or urine Angptl4 (*p* = 0.022, *p* = 0.032, respectively) (Figures 3C,D). Plasma Angptl4 or urine Angptl4 levels were positively correlated with urine protein levels (*r* = 0.310, *p* = 0.022; *r* = 0.340, *p* = 0.032, respectively). There was no significant correlation between the urine protein-creatinine ratio and plasma Angptl4 or urine Angptl4 levels (Figures 3E,F).

### Relationship of Plasma and Urine Angptl4 Levels With Podocyte Injury

**FIGURE 4:**
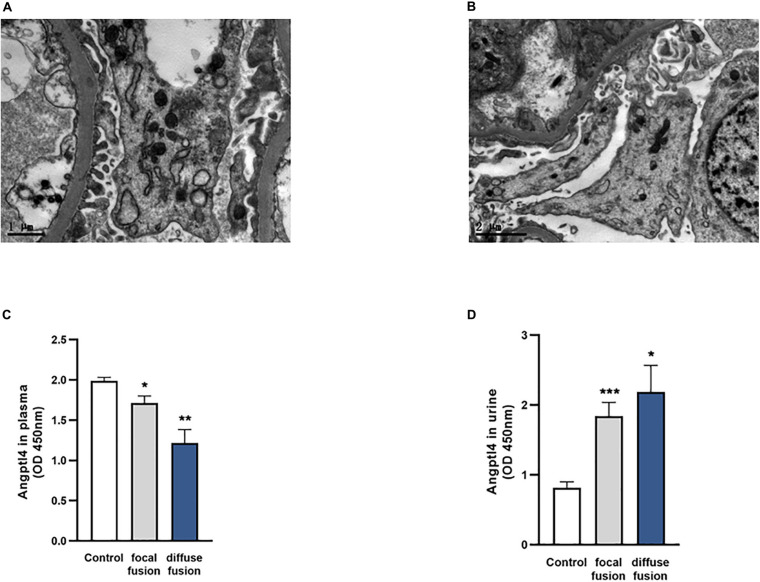
Analysis of podocyte foot process effacement in the two patient groups and the control group. **(A)** Electron microscopy image of no podocyte foot process effacement in the IgA nephropathy (IgAN) patients without podocyte group. **(B)** Electron microscopy image of podocyte foot process effacement in the IgAN patients with podocyte group (scale bar = 1 or 2 μm). **(C,D)** The expression of plasma and urinary Angptl4 in IgAN patients with podocyte foot process focal effacement (*n* = 28), diffuse effacement (*n* = 9), and controls (healthy and IgAN patients without podocyte injury) (*n* = 95). The data are expressed as the mean ± SEM. *P < 0.05, **P < 0.01, and ***P < 0.001 compared with the controls.

Among the 37 IgAN patients with podocyte injury, there were 28 patients with focal fusion of the foot process (<70%) and 9 patients with diffuse fusion of the foot process (≥70%). Representative electron microscopy images of IgAN patients with or without podocyte foot process effacement are shown in Figures 4A,B. The plasma Angptl4 levels in the focally fused group and the diffusely fused group were significantly lower than those in the controls (1.71 ± 0.09 versus 1.99 ± 0.04, *P < 0.05; 1.22 ± 0.17 versus 1.99 ± 0.04, **P < 0.01, respectively). There were no significant differences between the focally fused group and the diffusely fused group (Figure 4C). The Angptl4 level in the urine of the focally fused group was significantly higher than that in the control group (1.84 ± 0.19 versus 0.82 ± 0.08, ***P < 0.001; 2.19 ± 0.38 versus 0.82 ± 0.08, *P < 0.05, respectively). Similarly, there were no significant differences between the focally fused group and the diffusely fused group (Figure 4D).

## Discussion

A total of 20–40% of IgAN patients progress to ESRD by 20 years after biopsy, which significantly contributes to the population of patients with ESRD (Wyatt and Julian, 2013). Although progress has been made in understanding the pathogenesis of IgAN, predicting exactly which patients will progress to ESRD or how quickly they will progress remains a challenge. To identify the trend of the severe progression of patients as early as possible, to provide individualized and accurate treatment, and to delay the course of the disease has always been the aim of our research.

Proteinuria is one of the most important factors for assessing the risk of disease progression in patients with IgAN. Podocyte injury plays an important role in proteinuria during the severe progression of IgAN. Our previous studies found that Angptl4 affects podocytes and is much more sensitive and significant than circulating antibodies against PLA2R, a possible marker of idiopathic MN disease progression (Chen et al., 2013). Whether Angptl4 is related to podocyte damage in the progression of IgAN is the focus of this study.

Our previous studies found that the plasma level of LL-37, which is one of the ageing markers we identified (Jiang et al., 2008), is associated with the progression of human IgAN (Lu et al., 2014). Through bioinformatics analysis, we searched for aging-related genes that are also related to IgAN and found that *JUN* and *FOS* play an important role in the severe progression of IgAN, especially in the progression of fibrosis (Jiang et al., 2016). However, understanding and reversing the progression to the fibrotic stage early in this process remains a challenge.

In this study, we found that *FOS*, *MMP9*, and *G6PC* are hub genes that are related to both Angptl4 and podocytes through overlapping genes. *FOS* is an AP-1 transcription factor. Increased expression of AP-1 activates the RAAS system (Cao et al., 2013), which subsequently results in IgAN. *MMP-9* is a member of the matrix metalloproteinases (MMPs) and an important regulator of the extracellular matrix. MMP-9 plays a role in the decomposition of the mesangial matrix and mediates the recovery from pathogenic processes (Sekiuchi et al., 2012). G6PC (glucose-6-phosphatase catalytic subunit) is one of the three glucose-6-phosphatase catalytic subunit-encoding genes in humans. Mutations in this gene result in glycogen storage disease type I (GSD1), which is characterized by an ectopic accumulation of lipids in the liver and kidneys. Laure et al. showed that in K. G6pc^–/–^ mice, the renin-angiotensin system was activated, which caused increased Tgf-β1 expression, thereby activating epithelial-mesenchymal transition and subsequent fibrosis development (Clar et al., 2014).

To verify the potential roles of Angptl4 in IgAN, correlation analyses between Angptl4 levels and clinical features were performed. We found that the plasma and urine Angptl4 levels were strongly correlated with the degree of IgAN podocyte damage, which implies that Angptl4 may be a potential factor for evaluating IgAN progression in the future. Clement et al. (2011) showed that Angptl4 in urine is derived from a low-sialylated, high-pI, pro-proteinuric form that is secreted by podocytes, and its increase causes massive proteinuria. In this study, we also found that Angptl4 in urine is positively correlated with urine protein content. In contrast, in our experiment, Angptl4 in plasma was positively correlated with urine protein content, and (Clement et al., 2014) found that Angptl4 in plasma reduced proteinuria by binding to αvβ5 integrin. However, protein-creatinine ratio is not related to Angptl4 expression in plasma or urine.

In conclusion, the present study aimed to explore the possible functions of Angptl4 in IgAN progression. Three hub genes were screened via multiple-microarray analysis, and these genes may become potential targets for the diagnosis and treatment of IgAN in the future. However, this study has some limitations, as Angptl4 has an effect on podocyte damage in many kidney diseases, it is not specific to IgAN, and the size of biopsies of patients with IgAN used for expression was small. The underlying mechanism of podocyte damage is unclear. We further need to explore whether there is a connection between Angptl4 and the mechanism of telomere damage in aging, and we must conduct more thorough research regarding the molecular mechanism.

## Data Availability Statement

The sequencing data is deposited in the BioProject database (accession: PRJNA669346).

## Ethics Statement

The studies involving human participants were reviewed and approved by Ethical Committee of the Zhejiang University College of Medicine, the First Affiliated Hospital. The patients/participants provided their written informed consent to participate in this study.

## Author Contributions

SJ planned and conducted the project, collected the data, and wrote the manuscript. XP supplemented the project, analyzed the data, and wrote the manuscript. LL conducted the bioinformatics analysis. YZ and ML collected the sample. QZ conducted the IgAN pathologic diagnosis. XS conducted the Angptl4 experiment. YW processed the sample with the help of CW and SF. HJ designed and arranged the project with the help of JC and PR. HJ and PR provided the clinical support.

## Conflict of Interest

The authors declare that the research was conducted in the absence of any commercial or financial relationships that could be construed as a potential conflict of interest.

## References

[B1] Cao W. Xu J. Zhou Z. M. Wang G. B. Hou F. F. Nie J. (2013). Advanced oxidation protein products activate intrarenal renin-angiotensin system via a CD36-mediated, redox-dependent pathway. *Antioxid. Redox Signal.* 18 19–35. 10.1089/ars.2012.4603 22662869 PMC3503474

[B2] Chen W. Q. Zhang Y. Jiang H. Li H. Li X. Y. Yang X. (2013). Podocyte-related proteins in membranous nephropathy progression. *Chin. Med. J.* 126 3782–3783. 24112181

[B3] Clar J. Gri B. Calderaro J. Birling M. C. Hérault Y. Smit G. P. A. (2014). Targeted deletion of kidney glucose-6 phosphatase leads to nephropathy. *Kidney Int.* 86 747–756. 10.1038/ki.2014.102 24717294 PMC5678048

[B4] Clement L. C. Avila-Casado C. Mace C. Soria E. Bakker W. W. Kersten S. (2011). Podocyte-secreted angiopoietin-like-4 mediates proteinuria in glucocorticoid-sensitive nephrotic syndrome. *Nat. Med.* 17 117–122. 10.1038/nm.2261 21151138 PMC3021185

[B5] Clement L. C. Mace C. Avila-Casado C. Joles J. A. Kersten S. Chugh S. S. (2014). Circulating angiopoietin-like 4 links proteinuria with hypertriglyceridemia in nephrotic syndrome. *Nat. Med.* 20 37–46. 10.1038/nm.3396 24317117 PMC4114723

[B6] Faul C. Asanuma K. Yanagida-Asanuma E. Kim K. Mundel P. (2007). Actin up: regulation of podocyte structure and function by components of the actin cytoskeleton. *Trends Cell Biol.* 17 428–437. 10.1016/j.tcb.2007.06.006 17804239

[B7] Floege J. Feehally J. (2013). Treatment of IgA nephropathy and Henoch-Schonlein nephritis. *Nat. Rev. Nephrol.* 9 320–327. 10.1038/nrneph.2013.59 23545591

[B8] Fukuda A. Sato Y. Iwakiri T. Komatsu H. Kikuchi M. Kitamura K. (2015). Urine podocyte mRNAs mark disease activity in IgA nephropathy. *Nephrol. Dial. Transplant.* 30 1140–1150. 10.1093/ndt/gfv104 25956757 PMC4479668

[B9] Greka A. Mundel P. (2012). Cell biology and pathology of podocytes. *Annu. Rev. Physiol.* 74 299–323. 10.1146/annurev-physiol-020911-153238 22054238 PMC3600372

[B10] Hara M. Yanagihara T. Kihara I. (2007). Cumulative excretion of urinary podocytes reflects disease progression in IgA nephropathy and Schonlein-Henoch purpura nephritis. *Clin. J. Am. Soc. Nephrol.* 2 231–238. 10.2215/CJN.01470506 17699418

[B11] Hishiki T. Shirato I. Takahashi Y. Funabiki K. Horikoshi S. Tomino Y. (2001). Podocyte injury predicts prognosis in patients with iga nephropathy using a small amount of renal biopsy tissue. *Kidney Blood Press Res.* 24 99–104. 10.1159/000054214 11435741

[B12] Jiang H. Liang L. Qin J. Lu Y. Li B. Wang Y. (2016). Functional networks of aging markers in the glomeruli of IgA nephropathy: a new therapeutic opportunity. *Oncotarget* 7 33616–33626. 10.18632/oncotarget.9033 27127888 PMC5085107

[B13] Jiang H. Schiffer E. Song Z. Wang J. Zurbig P. Thedieck K. (2008). Proteins induced by telomere dysfunction and DNA damage represent biomarkers of human aging and disease. *Proc. Natl. Acad. Sci. U.S.A.* 105 11299–11304. 10.1073/pnas.0801457105 18695223 PMC2516278

[B14] Lai K. N. (2012). Pathogenesis of IgA nephropathy. *Nat. Rev. Nephrol.* 8 275–283. 10.1038/nrneph.2012.58 22430056

[B15] Lemley K. V. Lafayette R. A. Safai M. Derby G. Blouch K. Squarer A. (2002). Podocytopenia and disease severity in IgA nephropathy. *Kidney Int.* 61 1475–1485. 10.1046/j.1523-1755.2002.00269.x 11918755

[B16] Leung J. C. K. Lai K. N. Tang S. C. W. (2018). Role of mesangial-podocytic-tubular cross-talk in IgA nephropathy. *Semin. Nephrol.* 38 485–495. 10.1016/j.semnephrol.2018.05.018 30177020

[B17] Li J. S. Chen X. Peng L. Wei S. Y. Zhao S. L. Diao T. T. (2015). Angiopoietin-Like-4, a potential target of tacrolimus, predicts earlier podocyte injury in minimal change disease. *PLoS One* 10:e0137049. 10.1371/journal.pone.0137049 26352670 PMC4564140

[B18] Lu Y. Y. Yang X. Chen W. Q. Ju Z. Y. Shou Z. F. Jin J. (2014). Proteins induced by telomere dysfunction are associated with human IgA nephropathy. *J. Zhejiang Univ. Sci. B* 15 566–574. 10.1631/jzus.B1300115 24903994 PMC4116860

[B19] Ma J. Chen X. Li J. S. Peng L. Wei S. Y. Zhao S. L. (2015). Upregulation of podocyte-secreted angiopoietin-like-4 in diabetic nephropathy. *Endocrine* 49 373–384. 10.1007/s12020-014-0486-5 25424436

[B20] Sekiuchi M. Kudo A. Nakabayashi K. Kanai-Azuma M. Akimoto Y. Kawakami H. (2012). Expression of matrix metalloproteinases 2 and 9 and tissue inhibitors of matrix metalloproteinases 2 and 1 in the glomeruli of human glomerular diseases: the results of studies using immunofluorescence, in situ hybridization, and immunoelectron microscopy. *Clin. Exp. Nephrol.* 16 863–874. 10.1007/s10157-012-0633-3 22614167

[B21] Suzuki H. Kiryluk K. Novak J. Moldoveanu Z. Herr A. B. Renfrow M. B. (2011). The pathophysiology of IgA nephropathy. *J. Am. Soc. Nephrol.* 22 1795–1803. 10.1681/ASN.2011050464 21949093 PMC3892742

[B22] Wyatt R. J. Julian B. A. (2013). IgA nephropathy. *N. Engl. J. Med.* 368 2402–2414. 10.1056/NEJMra1206793 23782179

[B23] Yoshida K. Shimizugawa T. Ono M. Furukawa H. (2002). Angiopoietin-like protein 4 is a potent hyperlipidemia-inducing factor in mice and inhibitor of lipoprotein lipase. *J. Lipid Res.* 43 1770–1772. 10.1194/jlr.C200010-JLR200 12401877

